# A framework for assessing glaucoma progression using structural and functional indices jointly

**DOI:** 10.1371/journal.pone.0235255

**Published:** 2020-07-01

**Authors:** Sampson Listowell Abu, Iván Marín-Franch, Lyne Racette

**Affiliations:** 1 Department of Ophthalmology and Visual Sciences, University of Alabama at Birmingham, Birmingham, Alabama, United States of America; 2 Computational Optometry, Atarfe, Spain; University of California San Diego, UNITED STATES

## Abstract

**Purpose:**

While many tests and indices are available to identify glaucoma progression, using them in combinations may decrease overall specificity. The aim of this study was to develop a framework for assessing glaucoma progression using structural and functional indices jointly for a fixed specificity.

**Methods:**

The study included 337 eyes of 207 patients with ocular hypertension or primary open-angle glaucoma selected from the Diagnostic Innovations in Glaucoma Study or the African Descent and Glaucoma Evaluation Study. All patients had at least 9 visits. Each visit had retinal nerve fiber layer thickness (RNFLT) and mean sensitivity from static automated perimetry (SAP MS) measured within a one-month window. Simple linear regression was applied to assess deterioration in each index for series of 5 to 9 visits. To identify progression using the two indices jointly, marginal significance levels set at a specificity of 95% were derived for two criteria: ANY (worsening on either RNFLT or SAP MS) and ALL (worsening on both RNFLT and SAP MS). Positive rate (percentage of eyes flagged as progressing) was determined individually for each index, as well as for the ANY and ALL criteria.

**Results:**

Compared to SAP MS, RNFLT had higher positive rates (15% to 45%) for all series lengths. For the joint analyses, the positive rate was on average 12% higher for the ANY criterion compared to the ALL criterion. While RNFLT-alone had comparable positive rates and time-to-detection as the ANY criterion, each uniquely identified a subset of eyes (Kappa = 0.55 to 0.75).

**Conclusions:**

This study provides a simple framework for assessing glaucoma progression with data from two tests jointly, without compromising specificity. This framework can be extended to include two or more parameters, can accommodate global or regional indices, and can eventually be used with novel parameters identified as predictive of glaucoma progression.

## Introduction

Identifying glaucoma progression is essential to determine whether treatment should be modified to preserve vision and also to define the endpoints in clinical trials [1–3]. The detection of progression is challenging however [3], in part because of measurement variability [4–6] and in part because structural and functional assessments do not always agree [4, 7–9]. In addition, the lack of a known ground truth for glaucoma progression [10, 11] makes it impossible to evaluate with precision the sensitivities of different clinical tests. As an alternative, previous studies [11–13] have used the hit rate or positive rate to evaluate the performance of different tests at a fixed level of specificity, because arguably, a greater positive rate implies a greater sensitivity to detect progression.

Static automated perimetry (SAP) remains the clinical standard [2, 14], yet clinicians also rely on additional tests to monitor glaucoma progression: some based on retinal imaging [2, 15, 16] and others based on alternative forms of visual function testing [14, 17, 18]. For monitoring structural change in glaucoma, retinal nerve fiber layer thickness (RNFLT) measured either with scanning laser polarimetry [16] or optical coherence tomography (OCT) [19] has been shown to be more sensitive than neuro-retinal rim area (RA) obtained with confocal laser scanning ophthalmoscopy (CSLO). Furthermore, OCT has been reported to identify progression earlier than SAP in the early to moderate stages of glaucoma [20, 21]. Frequency doubling technology (FDT) perimetry has also been shown to identify progression earlier than SAP [22, 23]. Some reports, however, suggest that FDT and SAP have similar abilities to detect progression [14, 18, 24]. Despite the availability of these different tests, there is no agreement in the literature as to which tests are more sensitive to identify progression.

Although structural damage can precede functional loss in glaucoma, randomized clinical trials [7, 25, 26] and prospective cohort studies [9, 27, 28] have shown that functional deterioration can be detected in the absence of structural damage. Given that progression can be detected first on either structure or function in different eyes, it may be advantageous to assess both meticulously. Previous studies have investigated different approaches to combine structural and functional parameters with the goal of improving the detection of progression. Medeiros et al. developed a combined structure-function index which provides a percentage estimate of retinal ganglion cell loss [29]. Other attempts include statistical models that use structure and function measurements jointly to monitor glaucoma changes [30] and use structural information as a prior to estimate functional changes [31, 32]. In spite of these efforts, there is currently no consensus on how to use the information from multiple tests to identify change in glaucoma. The aim of this study was to develop a simple framework to assess glaucoma progression with structural and functional indices jointly.

## Methods

### Participants

We included 207 patients with ocular hypertension (OHT) or primary open-angle glaucoma (POAG) enrolled in the Diagnostic Innovations in Glaucoma Study (DIGS) or the African Descent and Glaucoma Evaluation Study (ADAGES). The DIGS and ADAGES are longitudinal studies designed to evaluate structure and visual function in healthy, OHT and POAG subjects at multiple study centers [33]. The DIGS and ADAGES followed the tenets of the Declaration of Helsinki and obtained the consent of all participants. The Institutional Review Board (IRB) at University of California, San Diego (UCSD) approved the DIGS study. The ADAGES was also approved by the IRBs at the 3 study centers: New York Eye and Ear Infirmary, University of Alabama at Birmingham (UAB) and UCSD. The current study was approved by the IRB at UAB. Eligibility included open anterior chamber angle, best-corrected visual acuity of 20/40 or better, spherical correction < 5 D and astigmatism < 3 D, one good-quality stereophotograph and one reliable visual field result. Participants with a history of intraocular surgery (except uncomplicated cataract or glaucoma surgery), secondary glaucoma, other ocular and systemic diseases that affect the visual field, cognitive impairment and inability to perform visual field tests reliably were not eligible. We adopted the classification scheme described in Sample et al [33] to classify participants at baseline. Participants with normal optic disc appearance but an intraocular pressure (IOP) ≥ 22 mmHg were classified as OHT patients. With or without elevated IOP, participants with 2 consecutive abnormal optic disc appearances and/or 3 consecutive abnormal visual fields were classified as POAG patients.

### Inclusion criteria for the current study

The present study included only participants who had 9 visits with OCT and SAP tests. At each visit, we required that OCT and SAP tests be taken no more than 30 days apart (both tests were taken on the same day in 73% of the visits and within a week in 87% of the visits). We also required that consecutive visits be separated by at least 2 months and by no more than 36 months.

### Structural assessment

RNFLT was measured with the Spectralis spectral domain OCT (software version 5.2.0.3, Heidelberg Engineering, Heidelberg, Germany). The Spectralis SD-OCT combines a dual-beam and CSLO to obtain high resolution RNFL circular scan. Each circular scan consisted of 1536 A-scans along a 3.45-mm circle centered on the optic nerve head [34]. Procedurally, pairs of thickness values from neighboring A-scans are averaged, and the resulting 768 RNFLT data points usually summarized as a global RNFLT. Sectorial RNFLT values were computed by averaging the thickness data points in each sector. The Imaging Data Analysis and Evaluation Reading Center at the Department of Ophthalmology, University of California, San Diego, assessed the quality of the OCT scans [33]. Only OCT scans with signal strength ≥ 15 dB and no artifacts were included [35]. When more than three good quality scans were taken on the same day, we randomly selected three scans and averaged them to obtain RNFLT.

### Functional assessment

All patients had a 24–2 SAP test at each visit. This was performed using the Swedish Interactive Thresholding Algorithm (SITA Standard) strategy on the Humphrey Field Analyzer II-i (Carl Zeiss Meditec Inc., Dublin, CA). The Visual Field Assessment Center at the Department of Ophthalmology, University of California, San Diego reviewed all visual fields for quality and reliability [36]. Only SAP tests with no artifacts and with less than 33% false positives (FP), false negatives (FN) and fixation losses (FL) were included in the present study. Approximately 98% of the 3,677 visual fields used in this study had < 15% FP and FN values and more than 86% had < 15% FL. The two locations above and below the blind spot were excluded from the analyses. To obtain the global mean sensitivity (SAP MS) values, the remaining 52 sensitivities were first converted from logarithmic (decibel, dB) into a linear scale, averaged as in Garway-Heath et al [37] and then converted back into decibel unit. Averaging the sensitivities in the linear scale minimizes overestimation of depth of defect as explained in Hood et al [38]. We also determined SAP MS values for the central region of the visual field by averaging the sensitivities for the 16 test points within the central region as described in Garway-Heath et al [37]. Likewise, SAP MS values were obtained for the infero-temporal (IT) and supero-temporal (ST) sectors as defined by the Garway-Heath map [39].

### Data analysis

Positive rates and 95% confidence intervals were obtained for the RNFLT and SAP MS individually and jointly. To assess progression with each index alone, we used simple linear regression to estimate the rate of change and computed the *t*-statistic and *p*-value for the one-tailed test that the slope is significantly negative (i.e., there is deterioration over time). This was done for each series length (5 to 9 visits). The specificity of the test was set at 95%, that is, the significance level was set to 0.05.

**Fig 1:**
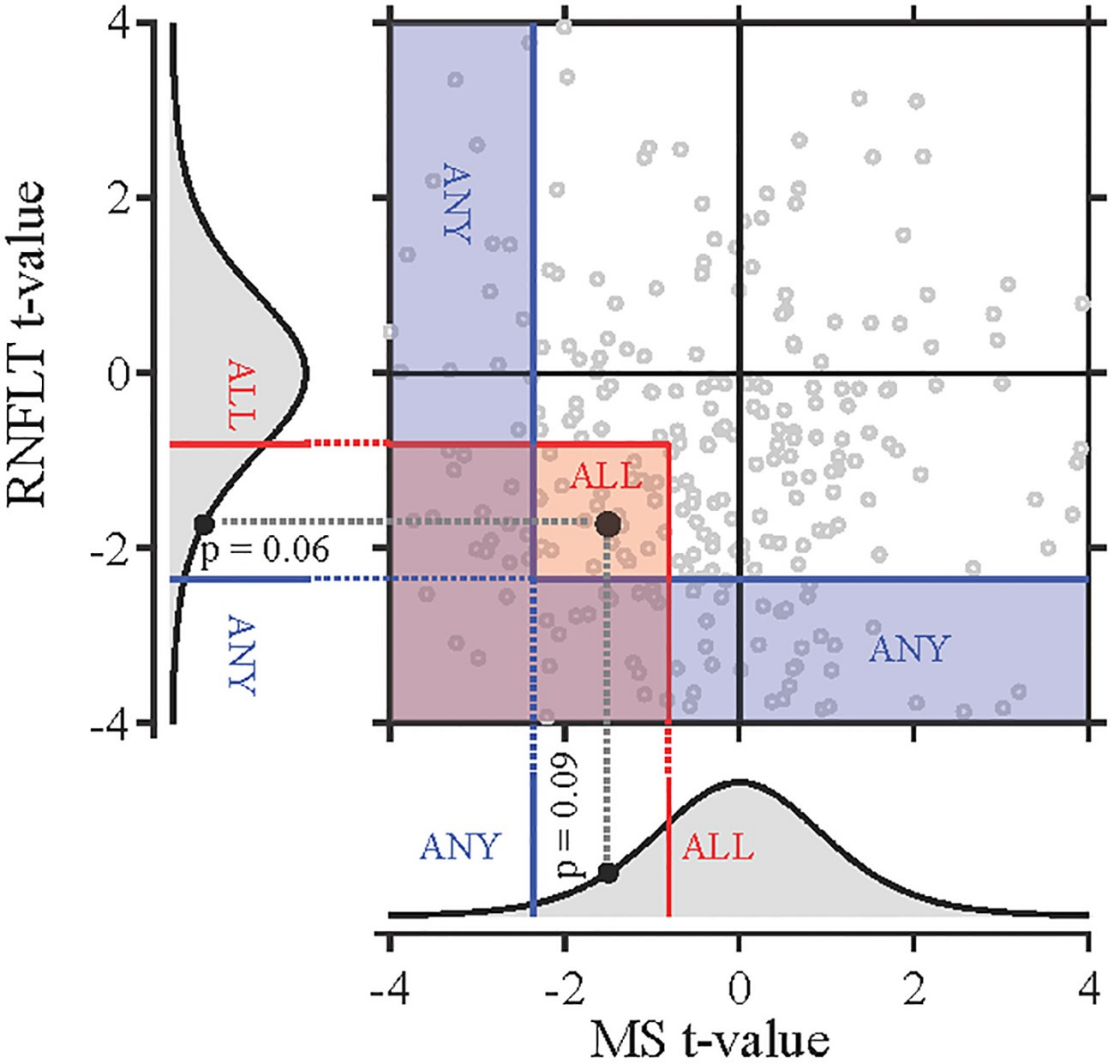
Illustration of the ALL and ANY criteria for progression with a structural (RNFLT) and a functional (SAP MS) index based on the one-tailed t-test that the slope is zero versus the slope is negative for simple linear regression. The individual null t-distributions are shown in the left (RNFLT) and bottom (SAP MS) panels. The critical region for the ALL criterion is demarcated with the red lines and the critical region for the ANY criterion is demarcated with the blue lines. Any pair of t-values in the area defined by the red lines would be classified as progressing by the ALL criterion. Any pair of t-values in the area defined by the blue lines would be classified as progressing by the ANY criterion. The open circles represent the joined t-values for individual eyes. The solid black circles are the observed t-values for RNFLT and SAP MS slopes derived from a series of 7 visits for an eye flagged as progressing with the ALL criterion for one eye. For clarity of presentation, the t-distribution range was restricted to t-values within– 4 to 4, which resulted in the exclusion of 80 eyes.

When progression was assessed using both indices jointly, we applied simple linear regression to each index individually and used two different criteria to determine the joint significance level. For the first criterion, an eye was flagged as progressing if the slopes for both RNFLT and SAP MS were significantly negative. We refer to this approach as the ALL criterion (worsening on both RNFLT and SAP MS). For the second criterion, an eye was flagged as progressing if the slope for either RNFLT or SAP MS was significantly negative. We refer to this approach as the ANY criterion (worsening on either RNFLT or SAP MS). Fig 1 illustrates these two progression criteria for using RNFLT and SAP MS jointly. To ensure a fair comparison between the positive rates for the individual indices and joint analyses, we fixed specificity at 95% by determining the marginal significance level needed for each joint criterion (see S1 Appendix). This is needed because using a significance level of 0.05 for the ALL and ANY criteria would result in specificities of 99.75%, and 89.75%, respectively. Thus, the ANY criterion, with its lower specificity, would appear to be more sensitive than the ALL criterion. Eyes were flagged as progressing when the joint p-values were smaller than 0.224 for the ALL criterion and when they were smaller than 0.025 for the ANY criterion.

Table 1 shows the marginal significance levels needed at three different levels of specificity, including the level used in this study (95%). Details on how to obtain marginal significance levels to fix specificity at any level for any number of functional and structural tests, and different progression criteria are given in the S1 Appendix. The level of agreement between individual indices and the joint assessment criteria was determined using the Kappa statistic (қ) [40]. With this statistic, agreement can be slight (0–0.20), fair (0.21–0.40), moderate(0.41–0.60), substantial (0.61–0.80), or almost perfect (0.81–1). The time-to-detection of progression was also compared between the individual and the joint progression criteria using Kaplan-Meier analysis [41]. All analyses were performed using R [42].

**Table 1:** Marginal significance levels for different progression criteria.

Progression criterion	Marginal Significance at different levels of specificity		
	90%	95%	99%
Individual index (e.g. RNFLT-alone, SAP MS-alone)	0.100	0.050	0.010
**ALL**: 2 indices (e.g., RNFLT *and* SAP MS)	0.316	0.224	0.100
**ANY**: 2 indices (e.g., RNFLT *or* SAP MS)	0.051	0.025	0.005

The marginal significance levels are shown for individual indices and pairs of indices at a set specificity of 90%, 95%, and 99%. (Note: In this study, we set specificity at 95%).

## Results

Participants demographic and disease characteristics are presented in Table 2. From the 207 patients, 337 eligible eyes (56 with OHT and 281 with POAG) were included in the analysis. The mean age and mean follow-up of this cohort were 64.3 ± 10.4 years and 4.4 ± 0.87 years, respectively.

**Table 2:** Demographic and disease characteristics of study participants.

Characteristics		
Mean age ± SD (years)		64.3 ± 10.4
Mean follow up time ± SD (years)		4.4 ± 0.87
Gender (N = 207)	*Female*	117 (56.5%)
	*Male*	90 (43.5%)
Race (N = 207)	*African descent*	90 (43.5%)
	*European descent*	102 (49.3%)
	*Others*	15 (7.2%)
Disease classification (N = 337 eyes)	*OHT*	56 (16.6%)
	*POAG*	281 (83.4%)
Disease severity (POAG eyes only)*	*Mild*	226 (80.4%)
	*Moderate*	33 (11.7%)
	*Advanced*	22 (7.8%)

SD = standard deviation

*Severity classification based on Hoddapp-Parrish-Anderson criteria [43].

**Fig 2:**
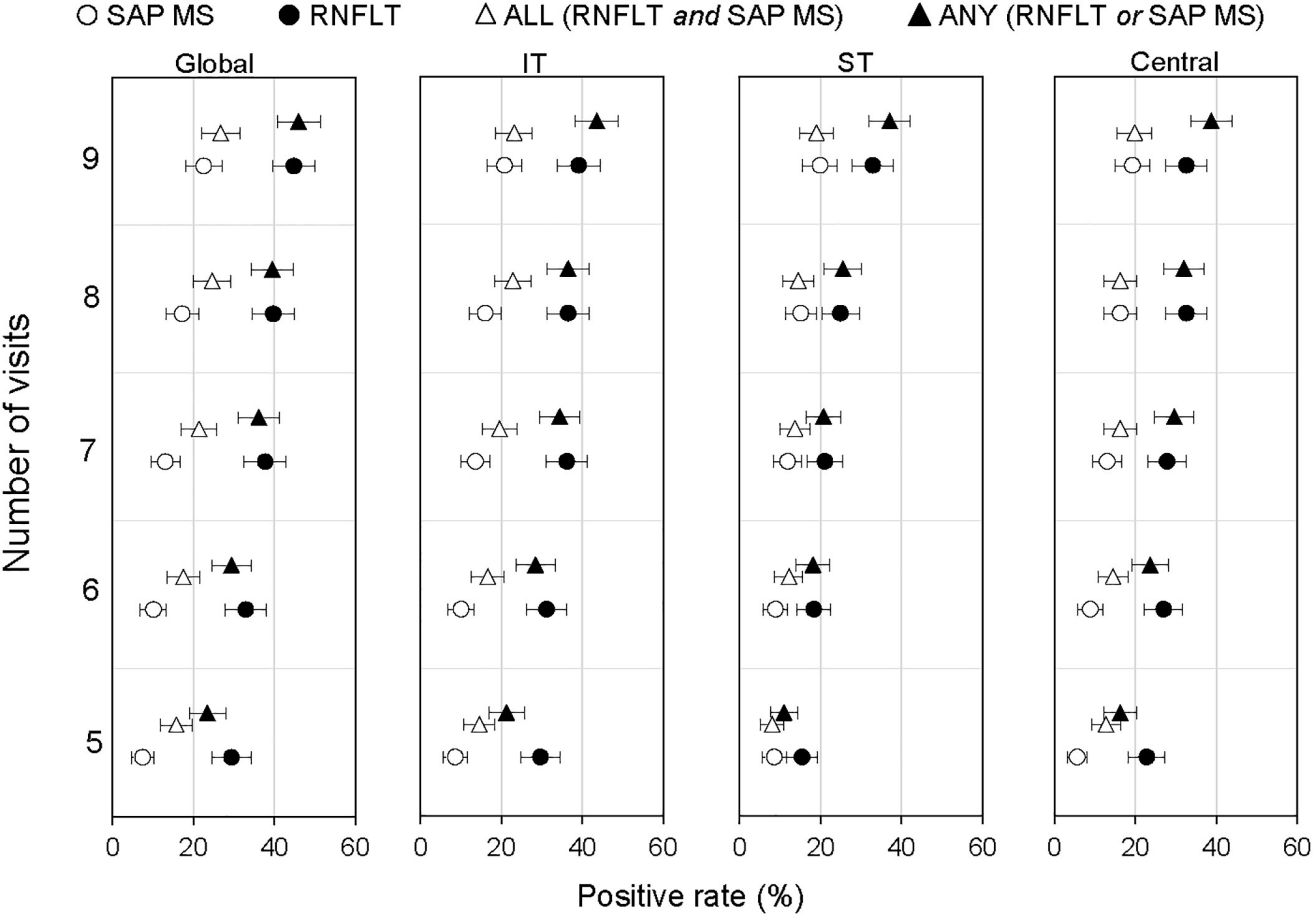
Positive rates and 95% confidence intervals for RNFLT-alone (solid circles), SAP MS-alone (empty circles), the ALL criteria (empty triangles) and the ANY criteria (solid triangles) for series of 5 to 9 visits. The data are presented for global, infero-temporal (IT), supero-temporal (ST), and Central region from the left to the right panels.

Fig 2 shows the positive rates obtained globally and in three sectors for RFNLT-alone, SAP MS-alone and for the different criteria used to assess progression with these 2 indices jointly. Positive rates for individual indices and for the joint analyses generally increased with series length as expected. For the individual indices, the positive rates for RNFLT-alone (10% to 45%) were higher than those observed for SAP MS-alone (5% to 23%). When RNFLT and SAP MS were analyzed jointly, the ANY criterion (worsening on either RNFLT or SAP MS) had higher positive rates (9% to 46%) compared to the ALL criterion (6% to 27%), with on average 10.6% more eyes flagged as progressing. The positive rates obtained for the ANY criteria were similar to those obtained for RNFLT-alone. Overall, the pattern of results was similar for the global and regional analyses.

**Fig 3:**
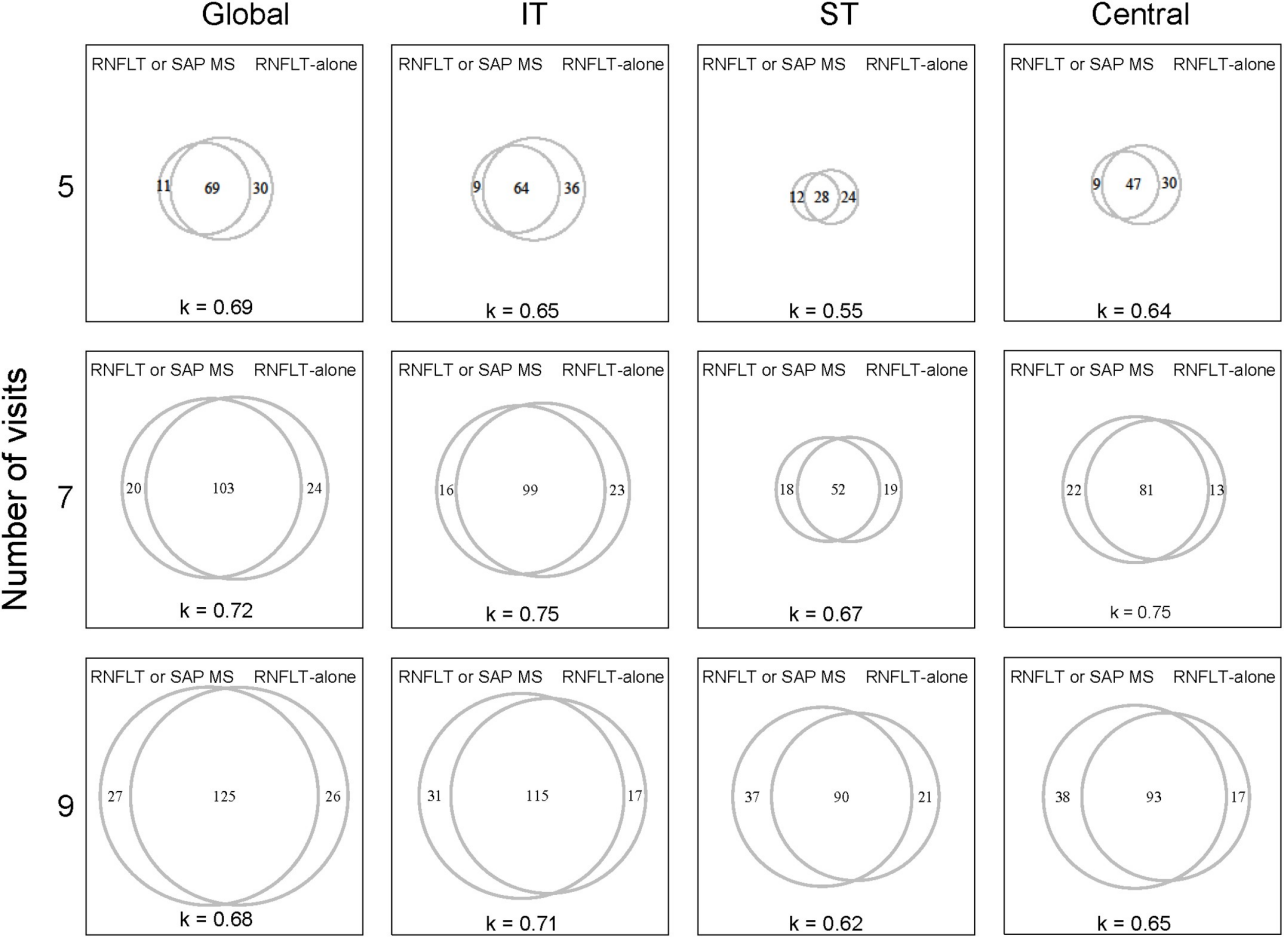
Proportional Venn diagrams illustrating the agreement between the eyes identified as progressing by RNFLT-alone and by the ANY criterion (RNFLT or SAP MS) for visits 5, 7 and 9. The Kappa statistic is shown at the bottom of each panel.

Although the joint assessments using the ANY criterion did not significantly improve the detection of progression compared to RNFLT-alone, different subset of eyes were identified as progressing. The proportional Venn diagrams shown in Fig 3 illustrate the agreement between the eyes identified as progressing with RNFLT-alone and the ANY criterion. There was moderate to substantial agreement between RNFLT-alone and the ANY criterion, with kappa values ranging 0.55 to 0.75. Depending on the number of visits and sector assessed, 13 to 27 eyes were uniquely identified as progressing with RNFLT-alone, whereas 9 to 38 eyes were uniquely identified by the ANY criterion. A similar analysis showed no agreement to slight agreement between SAP MS alone and RNFLT alone (қ = -0.07 to 0.04), with 13 to 47 eyes uniquely identified by SAP MS alone.

**Fig 4:**
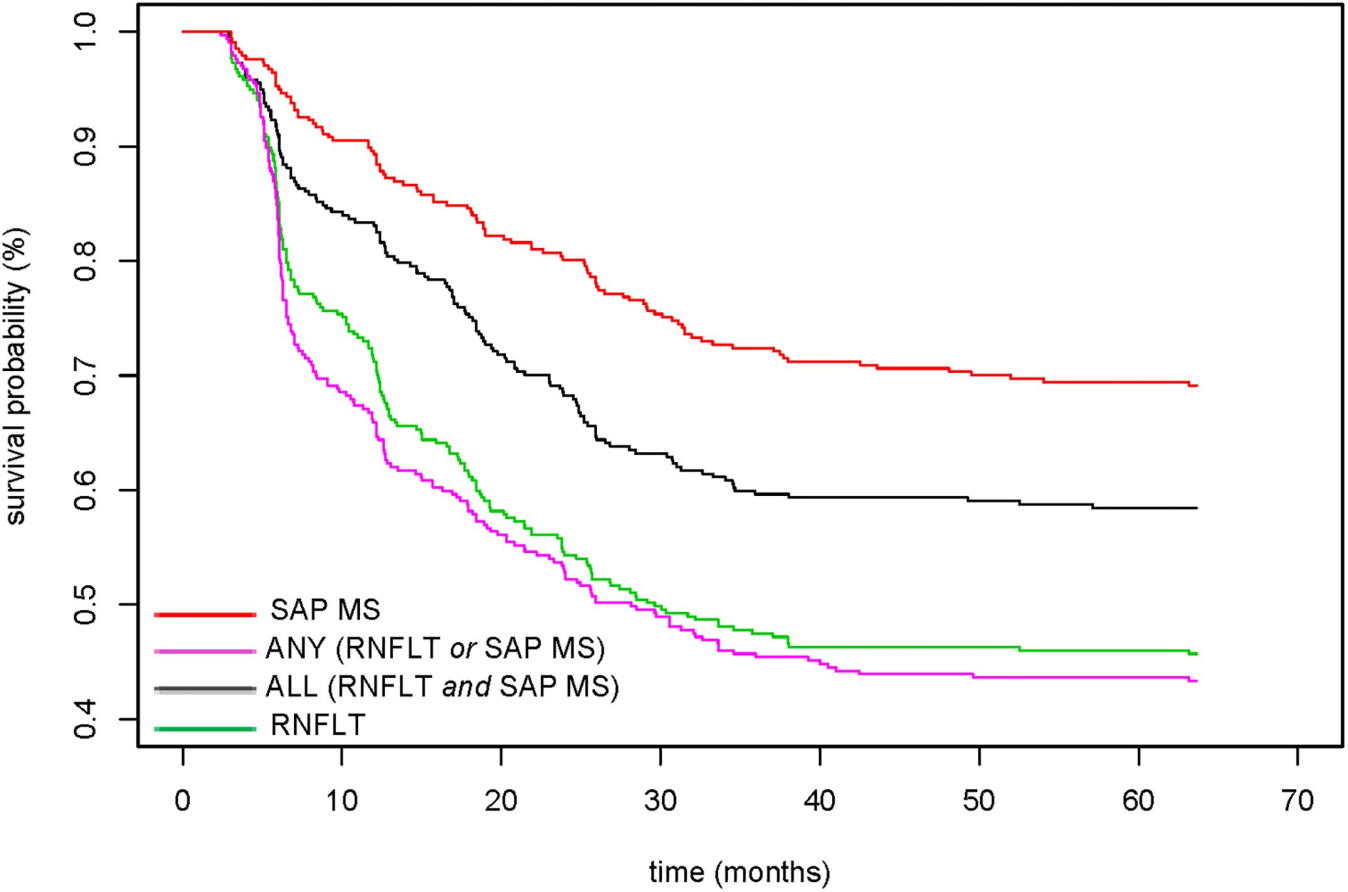
Estimated Kaplan-Meier survival curves for RNFLT and SAP MS as well as for the ALL and ANY criteria.

In addition to the positive rates, we were interested in assessing the time-to-detection of each joint criterion compared to each index alone. The results of the Kaplan-Meier analysis are shown in Fig 4. The median time-to-detection (time taken to detect 50% of the eyes as progressing) for both RNFLT-alone and the ANY (RNFLT or SAP MS) criterion was approximately 32 months. For this same period of time, SAP MS detected less than 25% of the eyes as progressing.

## Discussion

**Fig 5:**
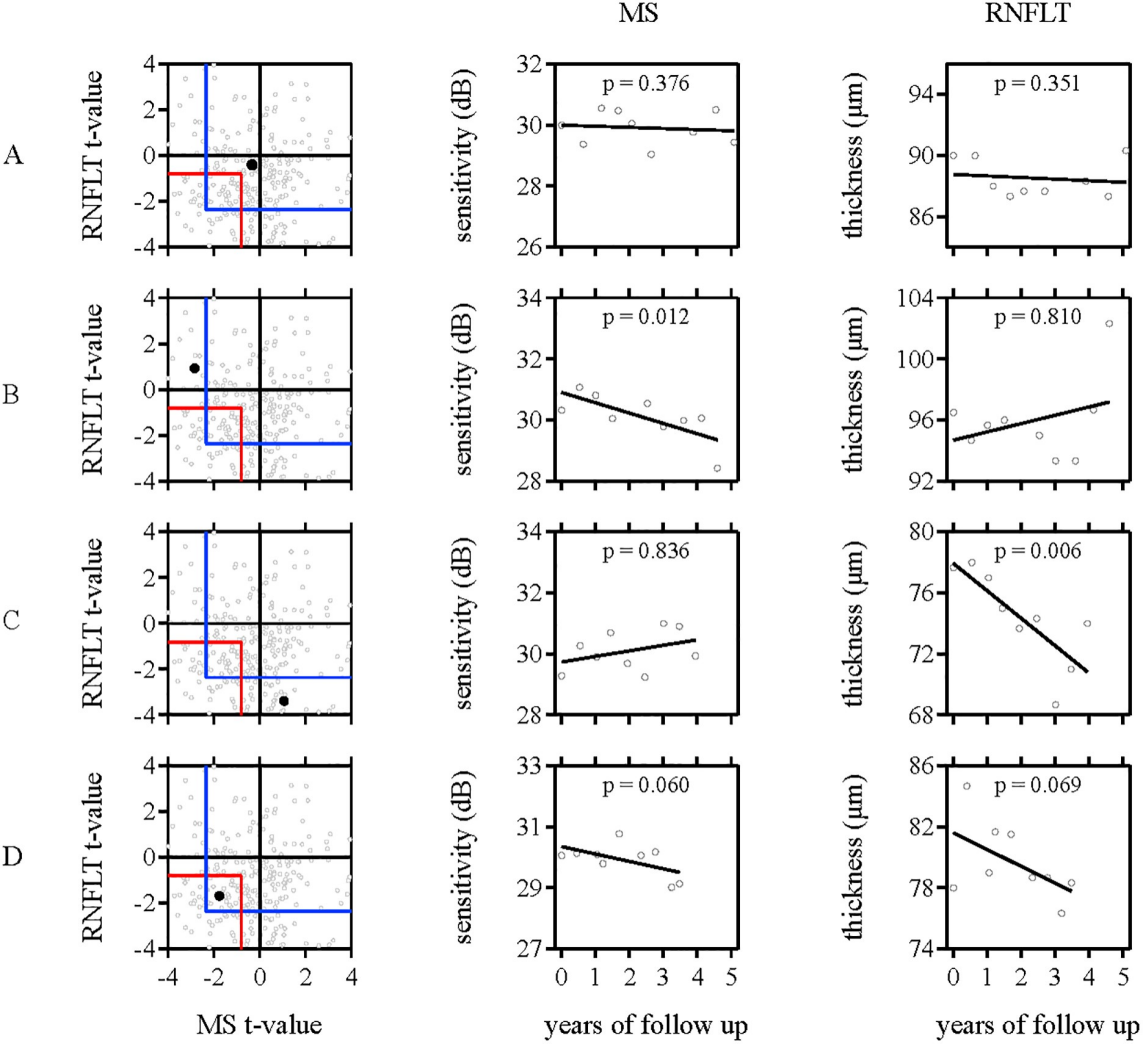
The SAP MS and RNFLT data are presented for four patients, along with the joint structural and functional assessment. When the slopes for both SAP MS and RNFLT are not statistically significant individually, the joint assessment can yield different outcomes depending on the direction and magnitude of the individual slopes. For example, the joint assessment of the patient in Panel A does not show progression, but that of the patient shown in Panel D shows progression. When there is disagreement between the longitudinal assessments of RNFLT and SAP MS, the ANY criteria will flag an eye as significant when a stricter marginal significance cut-off is reached for one of the measurements.

The framework developed in this study provides a formalized approach to evaluate the results of longitudinal structural and functional data jointly. The ALL and ANY progression criteria are similar in principle to the fundamental “all positive” and “either positive” rules that are commonly applied to analyze results from two clinical tests jointly [44]. To guard against increases in sensitivity that result from decreases in specificity when multiple tests are used jointly, we fixed specificity at 95% by determining the marginal significance level for each progression criterion. Fig 5 presents progression outcomes determined for four different patients to illustrate the different joint progression criteria. Panel A shows longitudinal data for a glaucoma patient who is not progressing. The individual slopes for RNFLT and MS are not significant and when considered jointly, the t-values do not reach statistical significance for the ANY and ALL criteria. In panel B, the individual longitudinal data from RNFLT and SAP MS disagree, with a significant slope for MS-alone and a non-significant slope for RNFLT. When considering both tests jointly, the t-value associated with the SAP MS p-value is large enough to exceed the cut-off value of the ANY criterion (see Table 1). A determination of progression can therefore be made for this patient. The example presented in panel C is similar, but with enough change observed for RNFLT. While the data for the patient presented in panel D shows only slight, non-significant, worsening on SAP MS and RNFLT individually, because there is worsening on both tests, progression is detected based on the ALL criteria when a joint assessment is performed.

The proposed framework requires marginal significance levels to be determined for the joint analysis of two (see Table 1) or more indices (S1 Appendix). This allows for as many parameters as desired to be considered jointly at a given level of specificity. The S1 Appendix explains how it is possible to group indices in different combinations (AND and OR) before applying the ANY and ALL criteria. For instance, information from 24–2 and 10–2 SAP tests can be analyzed jointly with peripapillary RNFLT and macula data from OCT. As new tests and parameters emerge in the future, this approach can be used to determine the optimal combination to identify glaucoma progression.

As mentioned in the Introduction, progression in different patients, can be detected first with either structural or functional measurements [26–28, 45]. The notion that using both types of measurements jointly may therefore improve the detection of glaucoma progression has led to the development of several strategies for joint structure-function analysis. The approach we propose in this study is similar to the Bayesian joint regression model developed by Medeiros et al [32], in that the identification of progression is based on the joint assessment of longitudinal structural and functional data. While the accuracy and precision of the slopes were assessed in that Bayesian model, its sensitivity was not evaluated. The framework we have proposed in this study can be used to perform such evaluations. Moreover, the framework is not limited to a specific definition of glaucoma progression. Besides the trend-based approach used in our study, other statistical methods of assessing progression, such as event-based [3] and permutation analyses [13], can be applied within this framework. For example, the outcomes of guided progression analysis from both OCT and SAP can be interpreted jointly with this framework at a desired level of specificity. A “possible progression” outcome on both devices may be considered a stronger indication of progression compared to a “likely progression” outcome from a single test.

Another model developed to assess progression using structural and functional data jointly is a complex multivariate mixed-effects model [46]. It was implemented within a Bayesian hierarchical modeling framework and identified progression in a significantly higher proportion of eyes (approximately 10% more eyes) compared to simple linear regression. In contrast, our results show that combining structure and function does not improve the detection of progression over using structure alone. This observation may have resulted from the application of a fixed specificity level, which ensured that the assessment of progression with RNFLT, alone or in combination with SAP MS, was held to the same level of statistical significance. Yousefi et al [47] applied various machine learning classifiers to structural and functional data individually and in combination. Similar to our findings, they reported that RNFLT-alone performs as well as the combination of RNFLT and SAP MS. Finally, in support of our finding that the joint analysis did not have a significant advantage over using structure alone, we observed that RNFLT alone and its combination with SAP MS had similar time-to-detection of progression (Fig 4).

Several studies [18, 48–50] have reported poor agreement between eyes identified as progressing by OCT and SAP indices. In the current study, the positive rates obtained for RNFLT-alone and the ANY criterion were approximately the same, however, different eyes were identified as progressing by each criterion (Fig 3). This is due to the detection of progression at different levels of significance between RNFLT-alone and the ANY criterion. As such, an eye that is identified as progressing by RNFLT-alone may not necessarily have the same outcome with the ANY criterion and vice versa. This finding suggests that when combining information from different tests to assess progression, it is also important to carefully consider the results of each test independently.

In clinical practice, SAP continues to be used as the preferred standard test for monitoring progression in moderate and advanced disease. The preference of SAP over OCT is often explained by the presence of a floor effect in RNFLT measurement with OCT [38, 51], a range beyond which no useful change in thickness can be detected. Recent studies, however, showed that whereas OCT was more sensitive for detecting progression in early glaucoma, its performance may not be significantly worse than SAP in moderate and advanced glaucoma eyes [20, 21]. Medeiros et al, however, showed that a single index generated by weighting structure and function based on disease severity was better at discriminating different disease stages [29]. This suggests that using structural and functional tests together to identify progression may perform differently across the spectrum of glaucoma severity. We compared positive rates between SAP MS, RNFLT and the ANY criterion in different glaucoma severities for the POAG eyes. The results from this analysis, presented in Table 3, show that using SAP MS alone or in combination with RNFLT may not perform better than using RNFLT alone in advanced disease. This implies that even in advanced disease, SAP may perform similarly to OCT to detect change. Comparable to the floor effect in RNFLT measurement with OCT, studies have shown that the ability of SAP to detect progression is limited when perimetric threshold estimates falls below 15–20 dB [52, 53]. Our analysis and that of previous studies [20, 21] involved a smaller sample of eyes with advanced disease. Further studies with larger samples of advanced glaucoma eyes are therefore needed to properly address whether SAP and OCT have different sensitivities in advanced disease.

**Table 3:** Positive rates and Kappa (k) agreement between RNFLT and ANY criterion in different severity of disease.

Visits	Mild (N = 226)				Moderate (N = 33)				Advanced (N = 22)			
	SAP MS (%)	RNFLT (%)	ANY (%)	k	SAP MS (%)	RNFLT (%)	ANY (%)	k	SAP MS (%)	RNFLT (%)	ANY (%)	k
**5**	7.10	30.50	23.50	0.73	9.09	24.24	18.18	0.46	13.64	9.09	9.09	0.45
**7**	14.60	39.40	38.90	0.71	18.18	36.36	30.30	0.73	9.09	9.09	13.64	0.78
**9**	24.80	44.70	48.20	0.68	18.18	42.42	42.42	0.63	18.18	27.27	18.18	0.49

The results reported herein should be considered in light of some limitations. First, our definition of progression was not adjusted for change due to normal aging. We did not correct for aging because, unlike mean deviation from SAP test, a conventional model to discount for age effects in RNFLT measurement is lacking. Moreover, the main focus of this study was to provide a framework to compare the detection of change by different test parameters, alone or in combination. Proposed strategies to adjust for aging, such as using the mean normal rate of change or the 5% lower limit as a reference [54], can be applied in our framework in future studies. The second limitation, a challenge for most progression detection methods, was the inability of all criteria to consistently identify the same eyes as progressing over time. We maintained a false positive rate of 5% for each progression criterion and across all series length, however, the between visit agreement presented in Table 4 shows that some eyes flagged as progressing at a particular visit were not flagged as such at subsequent visits, irrespective of the progression criterion applied. Because of the lack of a reference standard for glaucoma progression, some eyes may be identified as progressing when they are not (type I error), and other eyes may not be identified as progressing when in fact change is occurring (type II error). In addition, the variability present in the short follow-up data may account for the misidentification of eyes.

**Table 4:** Between-visit agreement (kappa) for eyes flagged as progressing by SAP MS-alone, RNFLT-alone, and the ANY criterion.

	SAP MS-alone	RNFLT-alone	ANY
**Visit 5 and 6**	0.45	0.69	0.63
**Visit 6 and 7**	0.71	0.74	0.69
**Visit 7 and 8**	0.69	0.79	0.73
**Visit 8 and 9**	0.66	0.76	0.81

In conclusion, several tests and parameters are currently available to monitor glaucoma progression. Each of these, as well as others that will be identified in the future, offer unique insights into glaucoma progression. The framework described in this study allows for many parameters to be considered jointly at fixed specificity levels. It is also compatible with different statistical methods to assess glaucoma progression. The framework allows the integration of different perspectives when monitoring glaucoma patients, allowing clinicians to jointly consider change on several clinically useful indices.

## Supporting information

S1 Appendix Derivation of marginal significance levels to fix specificity at any level for the joint analysis. (PDF)

S1 Dataset Dataset for 337 eyes containing only RNFLT and SAP MS measurements. (CSV)

## References

[1] Turpin A, Frank E, Hall M, Witten IH, Johnson CA. Determining progression in glaucoma using visual fields In: Cheung D, Williams GJ., Li Q. (eds) Advances in Knowledge Discovery and Data Mining. PAKDD 2001. Lecture Notes in Computer Science, Springer, Berlin, Heidelberg 2001.

[2] Brusini P. Monitoring glaucoma progression. Prog Brain Res. 2008;173:59–73. 10.1016/S0079-6123(08)01106-0 18929102

[3] Vianna JR, Chauhan BC. Chapter 7—How to detect progression in glaucoma. In: Bagetta G, Nucci C, editors. Prog Brain Res. 2015;221:135–58. 26518076 10.1016/bs.pbr.2015.04.011

[4] Malik R, Swanson WH, Garway-Heath DF. The ‘structure-function’ relationship in glaucoma—past thinking and current concepts. Clin Exp Ophthalmol. 2012;40(4):369–80. 10.1111/j.1442-9071.2012.02770.x 22339936 PMC3693944

[5] Chauhan BC, Johnson CA. Test-retest variability of frequency-doubling perimetry and conventional perimetry in glaucoma patients and normal subjects. Invest Ophthalmol Vis Sci. 1999;40(3):648–56. 10067968

[6] Heijl A, Lindgren A, Lindgren G. Test-retest variability in glaucomatous visual fields. Am J Ophthalmol. 1989;108(2):130–5. 10.1016/0002-9394(89)90006-8 2757094

[7] Öhnell H, Heijl A, Anderson H, Bengtsson B. Detection of glaucoma progression by perimetry and optic disc photography at different stages of the disease: results from the Early Manifest Glaucoma Trial. Acta Ophthalmologica. 2017;95(3):281–7. 10.1111/aos.13290 27778463 PMC5412870

[8] Hood DC, Kardon RH. A framework for comparing structural and functional measures of glaucomatous damage. Prog Retin Eye Res. 2007;26(6):688–710. 10.1016/j.preteyeres.2007.08.001 17889587 PMC2110881

[9] Artes PH, Chauhan BC. Longitudinal changes in the visual field and optic disc in glaucoma. Prog Retin Eye Res. 2005;24(3):333–54. 10.1016/j.preteyeres.2004.10.002 15708832

[10] Gardiner SK, Crabb DP. Examination of different pointwise linear regression methods for determining visual field progression. Invest Ophthalmol Vis Sci. 2002;43(5):1400–7. 11980853

[11] Garway-Heath DF, Quartilho A, Prah P, Crabb DP, Cheng Q, Zhu H. Evaluation of visual field and imaging outcomes for glaucoma clinical trials (An American Ophthalomological Society Thesis). Trans Am Ophthalmol Soc. 2017;115:T4 29085257 PMC5652981

[12] Zhu H, Russell RA, Saunders LJ, Ceccon S, Garway-Heath DF, Crabb DP. Detecting changes in retinal function: Analysis with non-stationary weibull error regression and spatial enhancement (ANSWERS). PLoS ONE. 2014;9(1):e85654 10.1371/journal.pone.0085654 24465636 PMC3894992

[13] O’Leary N, Chauhan BC, Artes PH. Visual field progression in glaucoma: estimating the overall significance of deterioration with permutation analyses of pointwise linear regression (PoPLR). Invest Ophthalmol Vis Sci. 2012;53(11):6776–84. 10.1167/iovs.12-10049 22952123

[14] Sample PA, Medeiros FA, Racette L, Pascual JP, Boden C, Zangwill LM, et al Identifying glaucomatous vision loss with visual-function–specific perimetry in the Diagnostic Innovations in Glaucoma Study. Invest Ophthalmol Vis Sci. 2006;47(8):3381–9. 10.1167/iovs.05-1546 16877406

[15] Kostanyan T, Wollstein G, Schuman JS. Evaluating glaucoma damage: emerging imaging technologies. Expert Rev Ophthalmol. 2015;10(2):183–95. 10.1586/17469899.2015.1012500 27087829 PMC4830491

[16] Alencar LM, Zangwill LM, Weinreb RN, Bowd C, Sample PA, Girkin CA, et al A comparison of rates of change in neuroretinal rim area and retinal nerve fiber layer thickness in progressive glaucoma. Invest Ophthalmol Vis Sci. 2010;51(7):3531–9. 10.1167/iovs.09-4350 20207973 PMC2904008

[17] Johnson CA. Psychophysical factors that have been applied to clinical perimetry. Vision Res. 2013;90:25–31. 10.1016/j.visres.2013.07.005 23872241

[18] Xin D, Greenstein VC, Ritch R, Liebmann JM, De Moraes CG, Hood DC. A comparison of functional and structural measures for identifying progression of glaucoma. Invest Ophthalmol Vis Sci. 2011;52(1):519–26. 10.1167/iovs.10-5174 20847115 PMC3053295

[19] Leung CK-S, Medeiros FA, Zangwill LM, Sample PA, Bowd C, Ng D, et al American Chinese Glaucoma Imaging Study: a comparison of the optic disc and retinal nerve fiber layer in detecting glaucomatous damage. Invest Ophthalmol Vis Sci. 2007;48(6):2644–52. 10.1167/iovs.06-1332 17525195

[20] Zhang X, Dastiridou A, Francis BA, Tan O, Varma R, Greenfield DS, et al Comparison of glaucoma progression detection by optical coherence tomography and visual field. Am J Ophthalmol. 2017;184:63–74. 10.1016/j.ajo.2017.09.020 28964806 PMC5894829

[21] Abe RY, Diniz-Filho A, Zangwill LM, Gracitelli CPB, Marvasti AH, Weinreb RN, et al The relative odds of progressing by structural and functional tests in glaucoma. Invest Ophthalmol Vis Sci. 2016;57(9):OCT421–OCT8. 10.1167/iovs.15-18940 27409501 PMC4968922

[22] Liu S, Yu M, Weinreb RN, Lai G, Lam DS-C, Leung CK-S. Frequency doubling technology perimetry for detection of visual field progression in glaucoma: a pointwise linear regression analysis. Invest Ophthalmol Vis Sci. 2014;55(5):2862–9. 10.1167/iovs.13-13225 24595388

[23] Hu R, Wang C, Racette L. Comparison of matrix frequency-doubling technology perimetry and standard automated perimetry in monitoring the development of visual field defects for glaucoma suspect eyes. PLoS ONE. 2017;12(5):e0178079 10.1371/journal.pone.0178079 28542536 PMC5436878

[24] Hu R, Wang C, Gu Y, Racette L. Comparison of standard automated perimetry, short-wavelength automated perimetry, and frequency-doubling technology perimetry to monitor glaucoma progression. Medicine. 2016;95(7):e2618 10.1097/MD.0000000000002618 26886602 PMC4998602

[25] Kass MA, Heuer DK, Higginbotham EJ, et al The Ocular Hypertension Treatment study: a randomized trial determines that topical ocular hypotensive medication delays or prevents the onset of primary open-angle glaucoma. Arch Ophthalmol. 2002;120(6):701–13. 10.1001/archopht.120.6.701 12049574

[26] Miglior S ZT, Pfeiffer N, Cunha-Vaz J, Torri V, Adamsons I; European Glaucoma Prevention Study (EGPS) Group. Results of the European Glaucoma Prevention Study. Ophthalmology. 2005;112(3):366–75. 10.1016/j.ophtha.2004.11.030 15745761

[27] Chauhan BC, McCormick TA, Nicolela MT, LeBlanc RP. Optic disc and visual field changes in a prospective longitudinal study of patients with glaucoma: comparison of scanning laser tomography with conventional perimetry and optic disc photography. Arch Ophthalmol. 2001;119(10):1492–9. 10.1001/archopht.119.10.1492 11594950

[28] Strouthidis NG, Scott A, Peter NM, Garway-Heath DF. Optic disc and visual field progression in ocular hypertensive subjects: detection rates, specificity, and agreement. Invest Ophthalmol Vis Sci. 2006;47(7):2904–10. 10.1167/iovs.05-1584 16799032

[29] Medeiros FA, Lisboa R, Weinreb RN, Girkin CA, Liebmann JM, Zangwill LM. A Combined index of structure and function for staging glaucomatous damage. Arch Ophthalmol. 2012;130(9):1107–16. 10.1001/archophthalmol.2012.827 23130365 PMC3787828

[30] Hu R, Marín-Franch I, Racette L. Prediction accuracy of a novel dynamic structure-function model for glaucoma progression. Invest Ophthalmol Vis Sci. 2014;55(12):8086–94. 10.1167/iovs.14-14928 25358735 PMC4266083

[31] Russell RA, Malik R, Chauhan BC, Crabb DP, Garway-Heath DF. Improved estimates of visual field progression using bayesian linear regression to integrate structural information in patients with ocular hypertension. Invest Ophthalmol Vis Sci. 2012;53(6):2760–9. 10.1167/iovs.11-7976 22467579 PMC4632869

[32] Medeiros FA, Zangwill LM, Girkin CA, Liebmann JM, Weinreb RN. Combining structural and functional measurements to improve estimates of rates of glaucomatous progression. Am J Ophthalmol. 2012;153(6):1197–205.e1. 10.1016/j.ajo.2011.11.015 22317914 PMC3804258

[33] Sample PA, Girkin CA, Zangwill LM, Jain S, Racette L, Becerra LM, et al The African Descent and Glaucoma Evaluation Study (ADAGES): design and baseline data. Arch Ophthalmol. 2009;127(9):1136–45. 10.1001/archophthalmol.2009.187 19752422 PMC2761830

[34] Silverman AL, Hammel N, Khachatryan N, Sharpsten L, Medeiros FA, Girkin CA, et al Diagnostic accuracy of the spectralis and cirrus reference database in differentiating between healthy and early glaucoma eyes. Ophthalmology. 2016;123(2):408–14. 10.1016/j.ophtha.2015.09.047 26526632 PMC4724549

[35] Miki A, Medeiros FA, Weinreb RN, Jain S, He F, Sharpsten L, et al Rates of retinal nerve fiber layer thinning in glaucoma suspect eyes. Ophthalmology. 2014;121(7):1350–8. 10.1016/j.ophtha.2014.01.017 24629619 PMC4310561

[36] Racette L, Liebmann JM, Girkin CA, Zangwill LM, Jain S, Becerra LM, et al African Descent and Glaucoma Evaluation Study (ADAGES): III. Ancestry differences in visual function in healthy eyes. Arch Ophthalmol. 2010;128(5):551–9. 10.1001/archophthalmol.2010.58 20457975 PMC2907156

[37] Garway-Heath DF, Holder GE, Fitzke FW, Hitchings RA. Relationship between electrophysiological, psychophysical, and anatomical measurements in glaucoma. Invest Ophthalmol Vis Sci. 2002;43(7):2213–20. 12091419

[38] Hood DC, Anderson SC, Wall M, Kardon RH. Structure versus function in glaucoma: an application of a linear model. Invest Ophthalmol Vis Sci. 2007;48(8):3662–8. 10.1167/iovs.06-1401 17652736

[39] Garway-Heath DF, Poinoosawmy D, Fitzke FW, Hitchings RA. Mapping the visual field to the optic disc in normal tension glaucoma eyes. Ophthalmology. 2000;107(10):1809–15. 10.1016/s0161-6420(00)00284-0 11013178

[40] Landis JR, Koch GG. The measurement of observer agreement for categorical data. Biometrics. 1977;33(1):159–74. 843571

[41] Kaplan EL, Meier P. Nonparametric estimation from incomplete observations. J Am Stat Assoc. 1958;53(282):457–81.

[42] R Core Team. R: A language and environment for statistical computing. Vienna, Austria: R Foundation for Statistical Computing; 2017.

[43] Hodapp E, Parrish RK II, Anderson DR. Clinical decisions in glaucoma. St Louis: The CV Mosby Co; 1993 pp. 52–61.

[44] Macaskill P, Walter SD, Irwig L, Franco EL. Assessing the gain in diagnostic performance when combining two diagnostic tests. Stat Med. 2002;21(17):2527–46. 10.1002/sim.1227 12205697

[45] Keltner JL, Johnson CA, Anderson DR, Levine RA, Fan J, Cello KE, et al The association between glaucomatous visual fields and optic nerve head features in the Ocular Hypertension Treatment Study. Ophthalmology. 2006;113(9):1603–12. 10.1016/j.ophtha.2006.05.061 16949445

[46] Medeiros FA, Leite MT, Zangwill LM, Weinreb RN. Combining structural and functional measurements to improve detection of glaucoma progression using Bayesian Hierarchical Models. Invest Ophthalmol Vis Sci. 2011;52(8):5794–803. 10.1167/iovs.10-7111 21693614 PMC3176049

[47] Yousefi S, Goldbaum MH, Balasubramanian M, Jung T-P, Weinreb RN, Medeiros FA, et al Glaucoma progression detection using structural retinal nerve fiber layer measurements and functional visual field points. IEEE Trans Biomed Eng. 2014;61(4):1143–54. 10.1109/TBME.2013.2295605 24658239 PMC4248722

[48] Wollstein G, Schuman JS, Price LL, Aydin A, Stark PC, Hertzmark E, et al Optical coherence tomography longitudinal evaluation of retinal nerve fiber layer thickness in glaucoma. Arch Ophthalmol. 2005;123(4):464–70. 10.1001/archopht.123.4.464 15824218 PMC1941777

[49] Na JH, Sung KR, Baek S, Lee JY, Kim S. Progression of retinal nerve fiber layer thinning in glaucoma assessed by Cirrus optical coherence tomography-guided progression analysis. Curr Eye Res. 2013;38(3):386–95. 10.3109/02713683.2012.742913 23441595

[50] Banegas SA, Antón A, Morilla-Grasa A, Bogado M, Ayala EM, Moreno-Montañes J. Agreement among spectral-domain optical coherence tomography, standard automated perimetry, and stereophotography in the detection of glaucoma progression. Invest Ophthalmol Vis Sci. 2015;56(2):1253–60. 10.1167/iovs.14-14994 25626965

[51] Mwanza J-C, Budenz DL, Warren JL, Webel AD, Reynolds CE, Barbosa DT, et al Retinal nerve fibre layer thickness floor and corresponding functional loss in glaucoma. Br J Ophthalmol. 2015;99(6):732–7. 10.1136/bjophthalmol-2014-305745 25492547 PMC4441828

[52] Wall M, Zamba GKD, Artes PH. The effective dynamic ranges for glaucomatous visual field progression with standard automated perimetry and stimulus sizes III and V. Invest Ophthalmol Vis Sci. 2018;59(1):439–45. 10.1167/iovs.17-22390 29356822 PMC5777662

[53] Gardiner SK, Swanson WH, Demirel S. The effect of limiting the range of perimetric sensitivities on pointwise assessment of visual field progression in glaucoma. Invest Ophthalmol Vis Sci. 2016;57(1):288–94. 10.1167/iovs.15-18000 26824408 PMC4736987

[54] Wu Z, Saunders LJ, Zangwill LM, Daga FB, Crowston JG, Medeiros FA. Impact of normal aging and progression definitions on the specificity of detecting retinal nerve fiber layer thinning. Am J Ophthalmol. 2017;181:106–13. 10.1016/j.ajo.2017.06.017 28669780

