# Supplementary material for: A framework for assessing glaucoma progression using structural and functional indices jointly
Source: PLoS One. 2020 Jul 1;15(7):e0235255. doi: 10.1371/journal.pone.0235255 (PMC7329074; doi:10.1371/journal.pone.0235255)
Supplement: S1 Appendix — (PDF) [file pone.0235255.s001.pdf]

Appendix A: Determining the marginal significance level for a set specificity

Let F and S be sets of slopes for a functional and a structural index that are lower than the estimated ones with OLS linear regression. Under the null hypothesis of no progression, the sets F and S are independent, and the probability that both true slopes for the functional and structural indices are smaller than those observed is

$$p_{\mathrm{ALL}} = P(F \cap S) = P(F)P(S) = \alpha_{\mathrm{ALL}}^2 \,,$$

where $\cap$ is the intersection between two sets. And the probability that either of the true slopes is smaller is

$$p_{\mathrm{ANY}} = P\{F \cup S\} = P(F) + P(S) + P(F \cap S) = 2\alpha_{\mathrm{ANY}} + \alpha_{\mathrm{ANY}}^2 \,,$$

where $\cup$ is the union between two sets. To achieve a false positive rate α —i.e., a specificity of $100(1-\alpha)\%$—, both p_{ALL} and p_{ANY} must equal α. Hence,

$$\begin{aligned} \alpha_{\mathrm{ALL}} &= \alpha^{1/2} \,, \\ \alpha_{\mathrm{ANY}} &= 1 - (1-\alpha)^{1/2} \,. \end{aligned}$$

For a false positive rate of 0.05, the marginal significance values for the ALL and ANY criteria with a structural and a functional index need be

$$\begin{aligned} \alpha_{\mathrm{ALL}} &= 0.224 \,, \\ \alpha_{\mathrm{ANY}} &= 0.0253 \,. \end{aligned}$$

This result can be generalized to an undetermined number of K indices I_k, with $k = 1, \cdots, K$. For the ALL criterion, the generalization is quite straightforward if we consider the sets $I_k^c, k = 1, \cdots, K$ of all velocity vectors that are greater than or equal to those observed. (The sets I_k^c are conjugate with respect to I_k.) Hence,

$$\alpha = P\left(\bigcap_{k=1}^{K} I_K\right) = \prod_{k=1}^{K} P(I_K) = \alpha_{\mathrm{ALL}}^K \,,$$

where $\prod$ is the product of sets' probabilities. The probability of the union equals the product of probabilities because the sets are independent under the null hypothesis of no progression. Therefore,

$$\alpha_{\mathrm{ALL}} = \alpha^{1/K} \,.$$

For the ANY criterion,

$$\begin{aligned} \alpha &= P\left(\bigcup_{k=1}^{K} I_K\right) = 1 - P\left(\bigcap_{k=1}^{K} I_K^c\right) \\ &= 1 - \prod_{k=1}^{K} P(I_K^c) = 1 - \prod_{k=1}^{K} \left[1 - P(I_K)\right] \\ &= 1 - (1 - \alpha_{\mathrm{ANY}})^K \,, \end{aligned}$$

and, therefore,

$$\alpha_{\text{ANY}} = 1 - (1-\alpha)^{1/K}\,.$$

For the combination of two functional and two structural indices, say F_1, F_2, S_1, and S_2 (e.g., SAP MS, FDP MS, RA, and RNFLT), the marginal significance values for the ALL and ANY criteria are

$$\begin{aligned}\alpha_{\text{ALL}} &= 0.473\,,\\ \alpha_{\text{ANY}} &= 0.0127\,.\end{aligned}$$

For 4 indices, we can think of many other criteria, depending on combination of pairwise comparisons. Two stand out given their clinical interpretation:

$$\begin{array}{ccc}(\mathrm{F_1\,OR\,F_2}) & \text{AND} & (\mathrm{S_1\,OR\,S_2})\,,\\ (\mathrm{F_1\,AND\,F_2}) & \text{OR} & (\mathrm{S_1\,AND\,S_2})\,.\end{array}$$

The first one (the AND criterion) can be interpreted as there is progression in at least one structural and one functional test. And since

$$\begin{aligned}\alpha &= P\left((F_1 \cup F_2) \cap (S_1 \cup S_2)\right)\\ &= P\left(F_1 \cup F_2\right) P\left(F_1 \cup F_2\right)\\ &= \left(1 - P(F_1^c)P(F_2^c)\right)\left(1 - P(S_1^c)P(S_2^c)\right)\\ &= \left[1-(1-P(F_1))(1-P(F_2))\right]\left[1-(1-P(S_1))(1-P(S_2))\right]\\ &= \left[1-(1-\alpha_{\text{AND}})^2\right]^2\,,\end{aligned}$$

then,

$$\alpha_{\text{AND}} = 1 - (1-\alpha^{1/2})^{1/2}\,.$$

The second one (the OR criterion) can be interpreted as there is confirmed progression in structure or function; confirmed in the sense that either both functional tests or both structural tests show progression. And since

$$\begin{aligned}\alpha &= P\left((F_1 \cap F_2) \cup (S_1 \cap S_2)\right)\\ &= \left[1 - P\left((F_1 \cap F_2)^c\right) P\left((S_1 \cap S_2)^c\right)\right]\\ &= \left[1 - P\left(F_1^c \cup F_2^c\right) P\left(S_1^c \cup S_2^c\right)\right]\\ &= \left[1 - (1 - P(F_1)P(F_2))\,(1-P(S_1)P(S_2))\right]\\ &= 1-(1-\alpha_{\text{OR}}^2)^2\,,\end{aligned}$$

then,

$$\alpha_{\text{OR}} = \left[1-(1-\alpha)^{1/2}\right]^{1/2}\,.$$

The marginal significance values for these two criteria are, respectively,

$$\begin{aligned}\alpha_{\text{AND}} &= 0.119\,,\\ \alpha_{\text{OR}} &= 0.159\,.\end{aligned}$$
